# Morin Disrupts Cytoskeleton Reorganization in Osteoclasts through an ROS/SHP1/c-Src Axis and Grants Protection from LPS-Induced Bone Loss

**DOI:** 10.3390/antiox11050963

**Published:** 2022-05-12

**Authors:** Hyun-Jung Park, Jung-Nam Park, Sun-Young Yoon, Rina Yu, Jae-Hee Suh, Hye-Seon Choi

**Affiliations:** 1 Department of Biological Sciences (BK21 Program), University of Ulsan, Ulsan 44610, Korea; oli_jjung@naver.com (H.-J.P.); elly1506@naver.com (J.-N.P.); tjsdud9981@naver.com (S.-Y.Y.); 2 Department of Food and Nutrition, University of Ulsan, Ulsan 44610, Korea; rinayu@ulsan.ac.kr; 3 Department of Pathology, Ulsan University Hospital, Ulsan 44030, Korea; drjhsuh1@gmail.com

**Keywords:** morin, inflammatory bone loss, osteoclast, Src homology region 2 domain-containing phosphatase 1, reactive oxygen species

## Abstract

Morin is a naturally occurring flavonoid with anti-inflammatory and antioxidative properties. Therefore, we hypothesized that morin may prevent inflammatory bone loss by reducing oxidative stress. To investigate the effect of morin on inflammatory bone loss, mice were injected with lipopolysaccharide (LPS). Osteoclasts (OCs) were analyzed by tartrate-resistant acid phosphatase (TRAP) staining and actin ring formation. Micro-computerized tomography analysis indicated that morin prevented LPS-induced bone loss in mice. In vivo TRAP staining indicated that morin decreased the number and surface of the OCs that were increased in LPS-treated mice. Furthermore, in vitro experiments indicated that morin decreased the number and activity of OCs upon LPS stimulation. Morin decreased actin ring-containing OCs with decreased activation of c-Src (Y416)/vav guanine nucleotide exchange factor 3/Ras-related C3 botulinum toxin substrate 1 compared with LPS alone. Morin decreased cytosolic reactive oxygen species (ROS), thus preventing the oxidation of Src homology region 2 domain-containing phosphatase 1 (SHP-1), followed by the inactivation of c-Src via direct interaction with SHP1. Conversely, SHP1 knockdown abolished the inhibitory effect of morin on OCs. Therefore, our findings suggest that morin disrupted cytoskeletal reorganization via an ROS/SHP1/c-Src axis in OCs, thereby granting protection from LPS-induced bone loss, which demonstrates its therapeutic potential against inflammatory bone loss.

## 1. Introduction

The occurrence of inflammatory diseases (e.g., rheumatoid arthritis, psoriatic arthritis, and Crohn’s disease) near bone tissues leads to focal erosion coupled with severe bone loss [1,2,3]. Moreover, inflammation has been reported to be positively associated with fracture risk [4]. Although osteoclasts (OCs) are bone-degrading cells that act as major effectors of inflammatory osteolysis [5], immune cells, mesenchymal cells, and their neighboring microenvironment also affect bone degradation through paracrine mechanisms when inflammation occurs. Excess IL-1β released by activated immune cells has been reported to enhance bone resorption directly [6]. Upregulation of receptor activator of nuclear factor kappa-B ligand (RANKL), tumor necrosis factor-α (TNF-α), and IL-17 have also been linked to bone loss by affecting bone cells [7]. Inflammation induces infiltrating immune cells to secrete various products to keep the inflammatory response, as well as initially recruit OCs, in turn later enhancing OC formation and activity. In animal models, LPS exhibits systemic inflammation, as well as irreversible bone loss, via enhancing OC activity [8,9,10]. Moreover, in vitro studies have shown that LPS affects OC through inducing OC differentiation and increasing its survival and function [11,12,13,14]. 

OC precursors are originated from hematopoietic cells and differentiate into bone-resorbing cells through several processes. Functional OCs are characterized by their unique morphological features. OC activation requires adhesion to the bone and subsequent cytoskeletal reorganization. The activity of OCs depends on the specialized cytoskeleton organization of an actin ring structure that forms the backbone of the bone resorption apparatus [15]. Therefore, given that actin is a major component of the cytoskeleton of OCs, actin-modulating factors act as key players for bone resorption. OCs express α_V_β_3_ integrin, which plays a critical role in cytoskeletal organization. The absence of α_V_β_3_ integrin has been demonstrated to impair cytoskeletal organization, resulting in increased bone density with disrupted bone resorption by OCs [16]. The tyrosine kinase c-Src is a downstream molecule of integrins that plays a critical role in forming a specialized actin ring for OCs [17], and the absence of c-Src results in severe osteopetrosis due to impaired OC function [18]. The c-Src/vav guanine nucleotide exchange factor 3 (Vav3) signaling complex has been reported to activate Ras-related C3 botulinum toxin substrate 1 (Rac1) for cytoskeletal reorganization, which is necessary for effective bone resorption [19,20,21]. 

Morin (3,5,7,20,4-pentahydroxyflavone) is a natural bioflavonoid that is isolated from *Maclura pomifera* [22]. This compound consists of two aromatic rings linked by a c-pyrone ring with multiple polar hydroxyl groups that possess free radical-scavenging properties [23]. Morin exhibits potent anti-inflammatory effects, as well as antioxidant activity, both in vitro and in vivo [24,25,26]. Morin has also been demonstrated to possess bone-sparing effects in glucocorticoid-induced bone loss [27].

In this study, we investigated the therapeutic potential of morin against LPS-induced bone loss in mice. Collectively, our findings provide insights into the molecular mechanisms through which morin attenuates LPS-induced cytoskeletal organization in OCs.

## 2. Materials and Methods

### 2.1. Ethics Statement

All mice were handled in accordance with the guidelines of the Institutional Animal Care and Use Committee (IACUC) of the Immunomodulation Research Center (IRC), University of Ulsan. All animal procedures were approved by the IACUC of the IRC. The approval ID for this study is #HSC-21-020.

### 2.2. Reagents and Antibodies

Morin was purchased from Tokyo Chemical Industry Ltd. (Tokyo, Japan). Recombinant mouse macrophage-colony stimulating factor (M-CSF) and receptor activator of nuclear factor κB ligand (RANKL) were obtained from R&D Systems, Inc. (Minneapolis, MN, USA). The assay kits for RatLaps EIA, osteocalcin EIA, alkaline phosphatase, and OxiSelectTM hydrogen peroxide were obtained from Immunodiagnostic Systems Inc. (Fountain Hills, AZ, USA), Biomedical Technologies Inc. (Stoughton, MA, USA), BioAssay Systems (Hayward, CA, USA), and Cell Biolabs Inc. (San Diego, CA, USA), respectively. LPS, MTT (3-(4,5-dimethylthiazol-2-yl)-2,5-diphenyltetrazolium bromide), leukocyte acid phosphatase (TRAP) assay kit, toluidine blue, *N*-acetyl-l-cysteine (NAC), and Hoechst 33258 were purchased from Sigma Chemical (St. Louis, MO, USA). Rhodamine phalloidin and *N*-(biotinoyl)-*N*′-(iodoacetyl) ethylenediamine (BIAM), Lipofectamine 3000, and Mito-SOX red were purchased from Molecular Probes (Carlsbad, CA, USA) and Invitrogen (Carlsbad, CA, USA), respectively. Antibodies against MCP-1 for coating and detection were obtained from R&D Systems, Inc. NE-PER nuclear and cytoplasmic extraction reagents were acquired from Pierce (Waltham, MA, USA). Regarding primary antibodies, antibodies specific for phospho-tyrosine (4G10) were purchased from Upstate USA Inc. (Charlottesville, VA, USA), and antibodies for Vav guanine nucleotide exchange factor 3 (Vav3) were obtained from Abcam (Cambridge, MA, USA). c-Src-Y416 and c-Src antibodies were obtained from Cell Signaling Technology (Denver, MA, USA) and ECM Biosciences (Versailles, KY, USA), respectively. β-Actin (A5441) and SHP1 (sc-287) antibodies were obtained from Sigma Chemical and Santa Cruz Biotechnology (Santa Cruz, CA, USA), respectively. Following the manufacturer’s protocol, active Rac1 was measured using the Rac1 activation kit from Thermo Scientific (Rockford, IL, USA). HRP-conjugated secondary antibodies and 2′,7′-dichlorofluorescein diacetate (H_2_DCFDA) were obtained from Thermo Fisher Scientific (Waltham, MA, USA). Small interfering RNA (siRNA) against SHP1 (sc-29479) and scrambled siRNA (scRNA, sc-37007) were obtained from Santa Cruz Biotechnology. M-MLV reverse transcriptase and SYBR Green Real-Time PCR Master Mixes were purchased from Promega (Madison, WI, USA). QIAzol reagent was purchased from Qiagen (Hilden, Germany). 

### 2.3. Animals and Study Design

Female C57BL/6J mice (10 weeks old) were housed in the IRC’s pathogen-free animal facility. Four groups of mice were randomly separated: vehicle control (*n* = 4), vehicle + morin 5 mg/kg (*n* = 4), LPS (*n* = 6), and LPS + morin 5 mg/kg (*n* = 6). LPS (5 mg/kg, i.p.) was injected into mice in 200 μL of phosphate-buffered saline (PBS) once a week for 3 weeks [28]. Morin was solubilized with DMSO and diluted with PBS to reach 0.75% DMSO. The mice were then intraperitoneally injected every day with morin in 200 μL of PBS (or just DMSO as a vehicle control) at a 5 mg/kg dose for 3 weeks. The mice were sacrificed in a CO_2_ chamber at the end of the treatment period. The femur was scanned using a high-resolution Micro CT (CT) SkyScan 1176 System (Bruker-Micro CT, Kontich, Belgium) to measure bone mineral density (BMD) and microarchitecture, as described in Park et al. [9]. Commercial RatLaps EIA test kits were used to determine the serum level of collagen-type I fragments (CTX-1). Serum levels of osteocalcin, alkaline phosphatase (ALP), and hydrogen peroxide were detected by an EIA specific for mouse osteocalcin kit, colorimetric kinetic determination kit, and a colorimetric H_2_O_2_ assay kit, respectively. Sandwich ELISA using the recommended antibodies was used to assess serum MCP-1, following the manufacturer’s instructions.

### 2.4. OC Formation

Bone marrow cells from 4–5 week old C57BL/6J mice were collected as described in a previous study [29], and downstream procedures were conducted as described by Park et al. [9]. In brief, the femora and tibiae were removed, and the marrow cavity was flushed out using a sterile 21-gauge needle. The cells were incubated in α-MEM containing 10% FBS in the presence of M-CSF (20 ng/mL). After 16 h, nonadherent cells were harvested and cultured for another 2 days. After washing twice, adherent monocyte/macrophage-like cells were treated with M-CSF (30 ng/mL) for growth and RANKL (40 ng/mL) for differentiation for 40 h. Pre-OCs were then incubated with M-CSF (30 ng/mL) and LPS (50 ng/mL) ± morin for 48 h to form OCs. Fully differentiated OCs were fixed in 10% formalin for 10 min and stained with tartrate-resistant acid phosphatase (TRAP). The total number of TRAP-positive multinucleated cells (MNCs) (three or more nuclei) was counted, and 70 cells were randomly selected for measuring area and maximum diameter. The fusion index was presented as the average number of nuclei per TRAP-positive MNCs [30].

### 2.5. Cell Viability

Bone marrow-derived macrophages (BMMs) were plated into 96-well plates. After generating pre-OCs, they were treated with LPS in the presence or absence of morin. MTT (3-[4,5-dimethyl-2-thiazolyl]-2,5-diphenyl-2*H*-tetrazolium bromide; 0.5 mg/mL) was used to assess viable cells at 37 ℃ for 3 h. After MTT removal, insoluble formazan products were dissolved in 100 μL of dimethylformamide (DMSO), and absorbance was measured using a microplate reader at 540 nm. 

### 2.6. RNA Isolation and Quantitative Polymerase Chain Reaction (qPCR)

Isolation of total RNAs was performed with QIAzol reagent, as described previously [9]. The expression of OC-specific genes was evaluated by qPCR analysis, and the 2^−ΔΔCT^ method was used to calculate relative mRNA expression. The expression level of OC-specific genes was normalized with housekeeping gene 18S rRNA (RPS). The primer sequences were 5′–CTC CAA CAA GGT GCT TGG GA–3′ and 5′–GAA GCA GTA GAT AGT CGC CA–3′ (calcitonin receptor); 5′–GAC CAC CTT GGC AAT GTC TCT G–3′ and 5′–TGG CTG AGG AAG TCA TCT GAG TTG–3′ (TRAP); 5′–GTG GGT GTT CAA GTT TCT GC–3′ and 5′–GGT GAG TCT TCT TCC ATA GC–3′ (cathepsin K); 5′–AGA CGT GGT TTA GGA ATG CAG CTC–3′ and 5′–TCC TCC ATG AAC AAA CAG TTC CAA–3′ (DC-STAMP); 5′–TTC AGT TGC TAT CCA GGA CTC GGA–3′ and 5′–GCA TGT CAT GTA GGT GAG AAA TGT GCT CA–3′ (ATP6v0d2); 5′–ATC AAT GCC AAC TAC GTG AAG AAC–3′ and 5′–GGC TGG CGA TGT AGG TCT TAGA–3′ (SHP1); and 5′–ATC AGA GAG TTG ACC GCA GTT G–3′ and 5′–AAT GAA CCG AAG CAC ACC ATA G–3′ (RPS).

### 2.7. Bone Resorption

To assess the bone resorption activity in vitro, mature OCs were generated with M-CSF for growth and RANKL for differentiation for 3–4 days in 24-well plates. Then, an equal number of obtained mature OCs were seeded on dentin discs [31]. The cells were then incubated with M-CSF (30 ng/mL) and LPS (50 ng/mL) in the presence or absence of morin (200 μM) for 4 days. Cells were fixed with formalin and stained for TRAP. To visualize resorption pits, cells were removed by 1 M NH_4_OH and stained with 1% (*w*/*v*) toluidine blue in 0.5% sodium borate. The resorbed area was measured through ImageJ software, 1.37 v.

### 2.8. Actin Cytoskeleton

To examine the actin ring within the OCs, mature OCs were washed and incubated under the indicated conditions for 6 h to examine filamentous polymer actin (F-actin) distribution as previously described [32]. OCs were fixed with formaldehyde on glass coverslips and incubated with 0.1% Triton X-100 in PBS for 5 min, followed by rhodamine phalloidin staining for actin and Hoechst 33258 for nuclei. The distribution of nuclei and F-actin was examined using an Olympus FV1200 confocal microscope (Olympus, Tokyo, Japan).

### 2.9. Western Blot Analysis

Total proteins from the cultured cells were extracted on ice with lysis buffer (50 mM Tris-HCl, pH 8.0, 150 mM NaCl, 1 mM EDTA, 0.5% Nonidet P-40, 0.01% protease inhibitor mixture), separated by SDS-PAGE, and transferred to nitrocellulose membranes. After blocking with 5% skim milk for 1 h at room temperature, the membranes were then incubated overnight with primary antibodies against c-Src (Y416), c-Src, Vav3, SHP1, and β-actin at 4 ℃. After washing with 1× TBS-T, the membranes were incubated with HRP-conjugated secondary antibodies for 1 h and developed using chemiluminescent substrates. 

### 2.10. Determination of Intracellular and Mitochondrial Reactive Oxygen Species (ROS)

Intracellular ROS and mitochondria-generated ROS were detected using the fluorescent probe 2′,7′-dichlorofluorescein diacetate (H_2_DCFDA). BMMs were prepared and incubated with M-CSF and RANKL for 40 h, washed thoroughly, incubated further for the indicated periods and conditions, harvested, suspended in PBS, loaded with H_2_DCFDA, and incubated at 37 °C for 30 min. Intracellular ROS were measured using an FACS Calibur flow cytometer (Becton Dickinson, Franklin Lakes, NJ, USA).

### 2.11. Detection of Oxidized SHP1 by Carboxymethylation

BMMs were incubated for 40 h with M-CSF (30 ng/mL) for growth and RANKL (40 ng/mL) for differentiation and further incubated with M-CSF and LPS in the presence or absence of morin (200 μM) for 16 h. The medium was then removed, and the cells were frozen rapidly in liquid nitrogen. The frozen cells were transferred to 100 µM *N*-(biotinoyl)-*N′*-(iodoacetyl) ethylenediamine (BIAM)-containing lysis buffer (50 mM Tris-HCl, pH 7.5, 150 mM NaCl, 0.5% Triton X-100, 10 µg/mL aprotinin, and 10 µg/mL leupeptin). The buffer was rendered oxygen-free by bubbling nitrogen gas through the buffer at a low flow rate for 20 min. The sulfhydryl-modifying chemical BIAM selectively detects the reduced form of cysteine [33]. After being sonicated three times in a water bath for 1 min, the lysate was clarified via centrifugation and subjected to immunoprecipitation with 200 µg of SHP1-specific antibodies. Immunocomplexes labeled with BIAM were detected with HRP-conjugated streptavidin, and the color was developed with an enhanced chemiluminescence kit.

### 2.12. Rac1 Activity Assay (Pulldown Assay)

The Rac1 pulldown assay was performed as indicated by the manufacturers. Briefly, cells were washed with PBS and harvested in the lysis buffer included in the kit. Next, 500 µg of cell lysates were mixed with GST–Pak1–PDB fusion protein in the presence of glutathione resin. The samples were then separated by SDS-PAGE (15% gel) and detected by immunoblotting using Rac1-specific antibodies.

### 2.13. Transfection of siRNA

After treatment with M-CSF and RANKL for 40 h, the BMMs were transfected with either a small interfering RNA (siRNA) against SHP1 or scrambled siRNA (scRNA) using Lipofectamine 3000. Downstream procedures were conducted as described by Park et al. [9]. 

### 2.14. Statistical Analyses

All experiments were repeated at least three times. The data are expressed as the mean ± standard deviation. Pairwise comparisons were conducted using Student’s *t*-test, whereas multiple group comparisons were conducted via one-way ANOVA followed by the Bonferroni post hoc test. Two-way ANOVA was used when two variables were analyzed. A *p*-value less than 0.05 was considered statistically significant. 

## 3. Results

### 3.1. Morin Protects Mice from LPS-Induced Bone Loss

**Figure 1:**
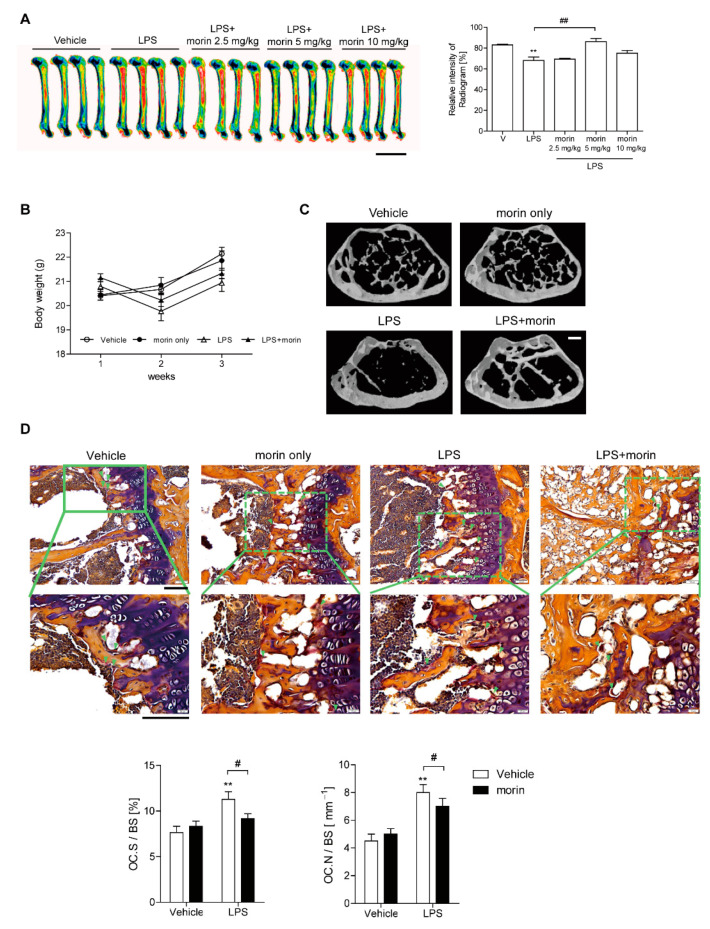
Protective effects of morin against LPS-induced bone loss in mice. (**A**) Representative images and relative intensities of X-ray radiograms of the distal femurs of mice treated with LPS (5 mg/kg/week) (*n* = 4), or LPS + morin (2.5 mg/kg/day, *n* = 4; 5 mg/kg/day, *n* = 4; 10 mg/kg/day, *n* = 4), which were measured using the Image J software. Scale bar: 5 mm. Mice began to be injected at 10 weeks of age. (**B**) Body weight changes during the treatment period. (**C**) Representative μCT images of distal femora 1.0 mm from the growth plate of mice treated with vehicle (*n* = 4), LPS (5 mg/kg/week) (*n* = 6), LPS + morin (5 mg/kg/day) (*n* = 6), or morin only (5 mg/kg/day) (*n* = 4). Scale bar: 2 mm. (**D**) Mouse femora were excised, cleaned, and decalcified in EDTA to analyze TRAP-positive OCs in vivo. Each of the four group’s histological sections were stained to indicate the presence of OCs (arrowhead) and calculate OC.S./BS (OC surface divided by total bone surface) and OC.N./BS (OC number divided by total bone surface). Image magnification: 200× (upper); 400× (lower). Scale bar: 100 μm. ** *p* < 0.01, compared with vehicle-treated mice; # *p* < 0.05, ## *p* < 0.01 compared with LPS-treated mice. Differences between groups were analyzed by two-way ANOVA, followed by Bonferroni post hoc tests to compare the effect of LPS (OC.S./BS, *p* < 0.05; OC.N./BS, *p* < 0.001), the effect of morin (OC.S./BS and OC.N./BS, not significant), and their interaction (OC.S./BS and OC.N./BS, not significant).

To investigate whether morin protected from inflammatory bone loss induced by LPS, µCT scans of femurs from mice injected with morin after LPS stimulation were analyzed. A 5 mg/kg dose of morin provided maximum protection compared to 2.5 and 10 mg/kg doses, as demonstrated by X-ray radiogram analysis (Figure 1A). There were no significant changes in body weight among the four groups at 13 weeks of age. Marked bone loss was observed upon LPS injection with decreased bone mineral density (BMD), bone volume (BV/TV), and trabecular thickness (Tb.Th), as well as increased trabecular spaces (Tb.Sp), compared to the vehicle control, as demonstrated by µCT analysis (Figure 1B and Table 1). In contrast, morin administration reversed the above-described effects induced by LPS (Figure 1B, Table 1). However, morin alone did not induce any significant differences compared with the vehicle control (Figure 1B, Table 1). In vivo TRAP staining demonstrated that LPS increased OC.S./BS (the ratio of OC surface to total bone surface area), whereas OC.N./BS (the ratio of OC number to total bone surface area) was significantly decreased upon injecting morin into LPS-treated mice (Figure 1C). Consistent with these observations, the expression of CTX-1, an in vivo bone resorption marker upregulated upon LPS injection, was dramatically reduced in the combined treatment of LPS and morin group. However, morin injection alone did not affect the expression of in vivo bone formation markers, serum ALP, or osteocalcin compared with LPS alone (Table 1). LPS alone increased the serum MCP-1 levels that were decreased by morin (Table 1). To determine whether morin attenuated inflammation-induced oxidative stress, serum ROS levels were also determined. LPS injection increased ROS levels, whereas combined treatment with morin significantly reduced them (Table 1).

### 3.2. Morin Reduces the Number of OCs upon LPS Stimulation In Vitro

**Figure 2:**
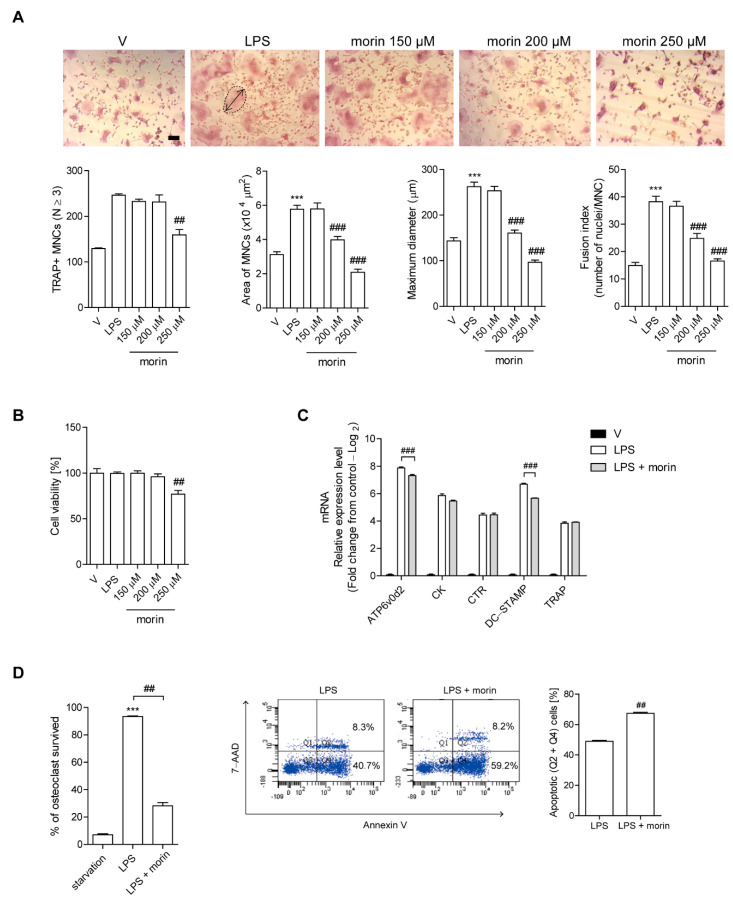
Morin reduces the number of OCs upon LPS stimulation in vitro. BMMs (10^4^ cells/well) were cultured with M-CSF (30 ng/mL) and RANKL (40 ng/mL) for 40 h, and then incubated further for 48 h (**A**,**B**) or 24 h (**C**) with M-CSF (30 ng/mL) and LPS (50 ng/mL) ± morin (150 μM, 200 μM, 250 μM). LPS and morin were dissolved in PBS and methanol, respectively, as a vehicle. (**A**) After fixation and TRAP staining, more than 70 MNCs were selected randomly to evaluate the OC area (dotted line) and the maximum diameter (double arrow). The fusion index is presented as the average number of nuclei per TRAP-positive MNCs formed in the culture. Representative photos are shown. Scale bar: 100 μm. (**B**) Cell viability was performed by the MTT assay, and no obvious toxicity was found for the range of used morin concentrations compared with vehicle-treated cells. (**C**) The mRNA expression of OC-specific genes in LPS-stimulated cells in the presence or absence of morin (200 μM) was analyzed by qPCR. Gene expression was visualized as the mean fold change of mRNA levels relative to RANKL pre-treatment samples (log_2_ scale). (**D**) Mature OCs were stimulated with LPS (50 ng/mL) with or without morin (200 μM) in the presence of M-CSF (30 ng/mL) for 6 h to measure TRAP-positive MNCs and annexin V-positive cells to evaluate OC survival. *** *p* < 0.001 compared with PBS-treated pre-OCs. ## *p* < 0.01, ### *p* < 0.001 compared with LPS-treated cells. Similar results were obtained from three independent experiments.

Since OCs played a critical role in the protective effect of morin against LPS-induced bone loss, as demonstrated by the data from in vivo TRAP staining, the effects of morin on OCs were investigated after LPS stimulation in vitro. Addition of LPS to RANKL-pretreated OC precursor cells induced their differentiation into OCs with maximum activation after 48 h of exposure, as evaluated by counting TRAP-positive MNCs. Morin reduced the surface area and maximum diameter of OCs and the fusion index that was calculated on the basis of the number of nuclei per OC in a concentration-dependent with a modest decrease in the number of TRAP-positive MNCs (Figure 2A). Furthermore, no significant changes in cell viability were observed except at the highest morin concentration (250 µM) under the assayed conditions; therefore, all downstream analyses were conducted using 200 µM of morin (Figure 2B). Our previous study showed that LPS induces the expression of the OC-specific genes TRAP, cathepsin K, DC-STAMP, ATP6v0d2, and calcitonin receptor in OCs [12], whereas morin decreased the mRNA expression of DC-STAMP and ATP6v0d2 without any change in TRAP, calcitonin receptor, or cathepsin K (Figure 2C). Since morin decreased the number of OCs without any changes in differentiation, we next sought to determine whether or not morin increased OC survival. Morin decreased the fraction of living OCs and increased annexin V-positive cells (Figure 2D).

### 3.3. Morin Inhibits LPS-Stimulated Actin Ring Formation and OC Activity

**Figure 3:**
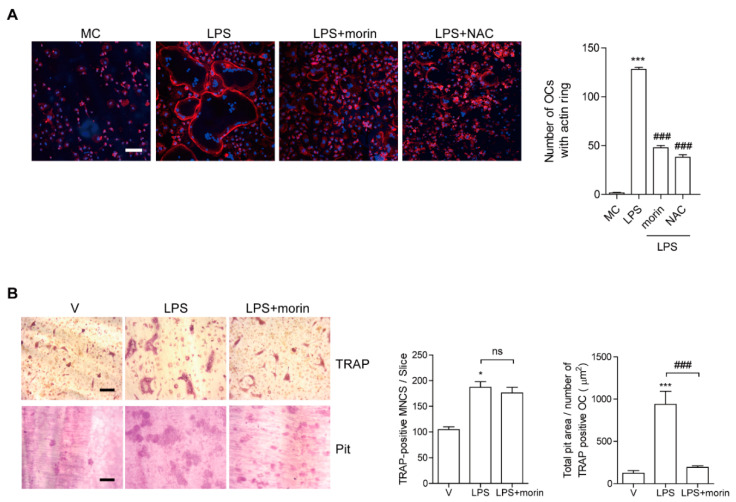
Morin inhibits LPS-stimulated actin ring formation and OC activity. BMMs were cultured with M-CSF (30 ng/mL) and RANKL (40 ng/mL) for 96 h. (**A**) Cells were incubated with α-MEM/10% FBS (medium control, MC) or with LPS (50 ng/mL) ± morin (200 μM) or NAC (3 mM) in the presence of M-CSF as indicated. After 6 h of incubation, the cells were stained with rhodamine phalloidin (red color) and Hoechst (blue color) to visualize actin rings and nuclei, respectively. Representative images are shown. Scale bar: 100 μm. The number of OCs with actin rings was plotted for the indicated conditions. (**B**) Mature OCs were seeded on dentine slices with M-CSF and LPS ± morin (200 μM) for 4 days. Cells were stained for TRAP to measure the number. After cells were removed, slices were stained for toluidine blue to calculate the resorption pits. Representative photos of TRAP-positive OCs and resorption pits are shown. Scale bar: 100 μm. The total pit area/number of TRAP-positive OCs was calculated. * *p* < 0.05, *** *p* < 0.001 compared with MC or vehicle; ### *p* < 0.001 compared with LPS. Similar results were obtained in three independent experiments.

Given that morin affected the surface area of OCs more dramatically compared with the number of OCs, we hypothesized that morin disrupts cytoskeleton reorganization in OCs. To evaluate whether morin decreases the actin ring formation necessary for bone resorption in OCs, mature OCs were generated on a plastic well and incubated with M-CSF and LPS in the presence or absence of morin, after which the cells were stained with rhodamine-phalloidin to visualize the actin ring. As shown in Figure 3A, no actin ring-containing OCs were observed upon depletion of M-CSF and LPS, whereas their addition increased the fraction of actin ring-containing cells. However, morin addition dramatically reduced it. When NAC, a ROS scavenger, was added to LPS-treated OCs, it also decreased it.

Next, in vitro bone resorption activity was evaluated using dentine slices to assess whether morin affected OC function. Mature OCs were generated from cells treated with morin in the presence of LPS. Combined treatment of morin lowered the total pit area/number of OCs in the LPS-treated cells (Figure 3B). 

### 3.4. Morin Decreases LPS-Induced Cytoskeletal Reorganization through a c-Src/Vav3/Rac1 Signaling Pathway in OCs

**Figure 4:**
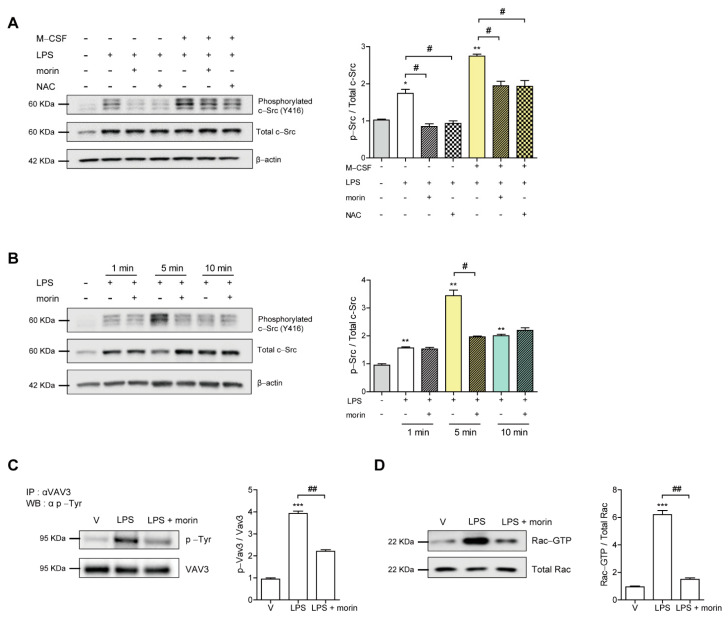
Morin decreases LPS-induced cytoskeletal reorganization via a signaling pathway involving c-Src/Vav3/Rac1in OCs. BMMs were cultured with M-CSF (30 ng/mL) and RANKL (40 ng/mL) for 96 h. Cells were washed with PBS and stimulated with LPS (50 ng/mL) ± morin (200 μM) or NAC (3 mM) in the presence or absence of M-CSF (30 ng/mL) as indicated. As a control, BMM was loaded at the first lane. The cell lysate was immunoblotted for phosphorylated c-Src (Y416) after 5 min of exposure (**A**) or the indicated timepoints (**B**). The strong central band was calculated for phosphorylated c-Src. After 5 min of exposure, phosphorylation of Vav3 on tyrosine residue was detected by immunoprecipitation with anti-Vav3 and anti-phosphorylated Tyr (**C**). GST pulldown and immunoblotting with Rac-specific antibodies were used to assess GTP-Rac (**D**). * *p* < 0.05, ** *p* < 0.001, *** *p* < 0.001 compared with vehicle; # *p* < 0.05, ## *p* < 0.01 compared with LPS-treated cells. Similar results were obtained in three independent experiments.

To investigate the mechanisms through which morin decreases the occurrence of actin-containing OCs, we next sought to characterize the effect of morin on cytoskeletal reorganization in OCs. M-CSF has been reported to collaborate with α_V_β_3_ integrin to activate canonical signaling pathways consisting of c-Src, Vav3, and Rac1, which enable the cells to form actin rings [34]. Next, we determined whether the addition of LPS enhanced the activation of c-Src induced by M-CSF alone. As shown in Figure 4A, combined treatment of LPS and M-CSF enhanced the level of phosphorylated c-Src (Y416). LPS in the presence of M-CSF exhibited the maximum level of phosphorylated c-Src (Y416) after 5 min of exposure, whereas the combined treatment of LPS and morin attenuated it dramatically (Figure 4B). Next, we assessed whether lowered c-Src activation by morin attenuated the activation of Vav3 and Rac1 to disrupt actin ring formation induced by LPS and M-CSF. Consistent with its effect on c-Src, morin decreased level of phosphorylated Vav3 at Tyr residue stimulated upon M-CSF and LPS (Figure 4C,D). Activation of Rac1 was evaluated by a glutathione *S*-transferase (GST) pulldown assay. While activated form of Rac1-GTP was found after 5 min of exposure to M-CSF and LPS, the addition of morin attenuated its activation (Figure 4C). Consistent with its morphological and functional phenotypes (Figure 3A), morin disrupted the major cytoskeleton-organizing signals through a decrease in c-Src/Vav3/Rac1 signaling in OCs.

### 3.5. Morin Decreases LPS-Induced Oxidation of SHP-1 to Attenuate c-Src Activation by Decreasing ROS Levels in OCs

**Figure 5:**
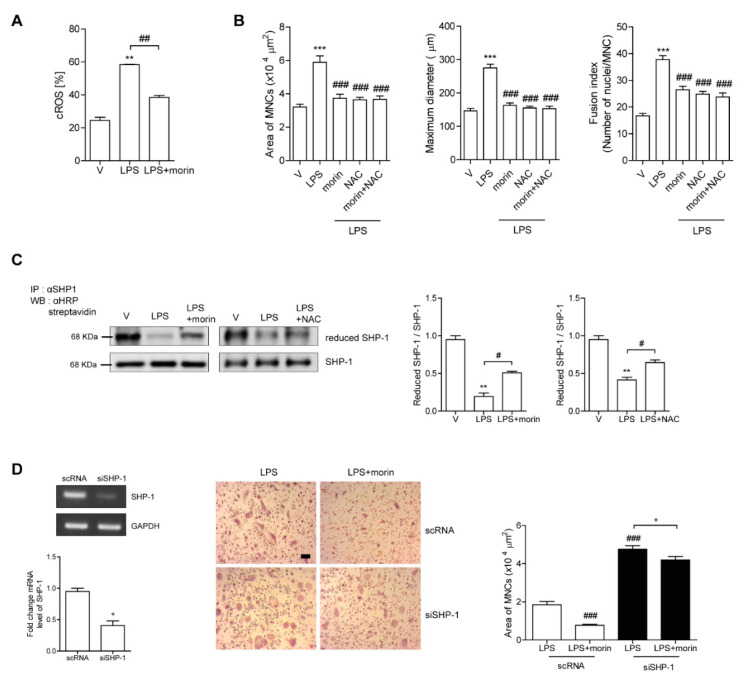
Morin decreases LPS-induced oxidation of SHP-1 to attenuate c-Src activation by decreasing the ROS level in OCs. (**A**) BMMs were cultured with M-CSF (30 ng/mL) and RANKL (40 ng/mL) for 96 h. Cells were washed with PBS and stimulated with LPS (50 ng/mL) ± morin (200 μM) in the presence of M-CSF (30 ng/mL). Cytosolic ROS were determined after 6 h exposure of LPS ± morin (200 μM) in the presence of M-CSF by flow cytometry using H_2_DCF-DA (B-D). Cells were incubated with M-CSF (30 ng/mL) and RANKL (40 ng/mL) for 40 h, and then further stimulated with M-CSF and LPS (50 ng/mL) ± morin (200 μM) or NAC (3 mM) for 48 h. More than 70 TRAP-positive MNCs randomly selected, and the surface area, maximum diameter, and fusion index of OCs were plotted (**B**). Immunoprecipitation (IP) with anti-SHP1 was performed after labeling the cell lysate with *N*-(biotinoyl)-*N*′-(iodoacetyl) ethylenediamine (BIAM), followed by immunoblotting with HRP-streptavidin to identify the reduced form of SHP1 as shown (**C**). The cells were transfected with scRNA or siSHP1 and incubated further with LPS (50 ng/mL) ± morin (200 μM) in the presence of M-CSF (30 ng/mL) for 48 h. The downregulation of SHP-1 by siRNA was confirmed by RT-PCR and qPCR. The cells were fixed, and the surface area of the OCs was measured (**D**). Representative photos are shown. Scale bar: 100 μm. ** *p* < 0.001, *** *p* < 0.001 compared with vehicle; # *p* < 0.05, ## *p* < 0.01, ### *p* < 0.001 compared with LPS-treated cells; ^+^ *p* < 0.05 compared with each corresponding control. Similar results were obtained from three independent experiments.

To investigate the mechanisms via which morin decreases c-Src activation in OCs, we investigated whether LPS-induced ROS played a role in modulating c-Src activation. Morin markedly decreased cROS after 6 h of exposure to LPS in mature OCs, as shown in Figure 5A. When NAC was added to LPS-treated OCs, phosphor-cSrc (Y416) was significantly attenuated (Figure 4A). NAC also reduced the occurrence of LPS-induced actin ring-containing OCs (Figure 3A). In LPS-induced OCs, there was no additive effect on the surface area, maximum diameter, and fusion index of OCs by adding both NAC and morin together (Figure 5B), suggesting that they work through the same pathway and not two different pathways. 

Given that SHP-1 is a nonreceptor-type protein tyrosine phosphatase that may decrease c-Src (Y416) phosphorylation [35], and that our previous studies demonstrated a direct interaction between c-Src and SHP-1 upon ROS stimulation [36], we hypothesized that decreased c-Src activation by morin was mediated by increased active SHP-1 due to decreased ROS levels. Next, we assessed whether morin reduced the oxidized form of SHP-1 to attenuate c-Src activation. As shown in Figure 5C, LPS decreased the level of reduced SHP-1, which in turn increased the levels of phosphorylated c-Src (Y416). In contrast, morin recovered the levels of the reduced form of SHP-1 and decreased phosphorylated c-Src (Y416) in the presence of LPS. Interestingly, NAC induced the same pattern as morin. To confirm the role of SHP-1 in the effect of morin on OCs, knockdown of SHP-1 was performed in OCs. Silencing of SHP-1 attenuated the decreasing effect of morin on the surface area of OCs (Figure 5D). Furthermore, the increased surface area of OCs upon LPS stimulation was enhanced by knockdown of SHP-1 compared to the scRNA-treated control (Figure 5D).

## 4. Discussion

Our findings demonstrated that morin, a natural bioflavonoid, protected mice against LPS-induced inflammatory bone loss. Three weeks after LPS injection, the mice exhibited a marked reduction in bone mineral density, which was accompanied by an increase in the number and surface area of OCs without affecting the expression of in vivo bone formation markers, suggesting that OCs play critical roles in LPS-induced bone loss. Morin injection reduced the number and surface area of OCs, as well as serum CTX-1 levels, which were enhanced in LPS-treated mice. No changes in serum ALP and osteocalcin were observed, indicating that the bone-sparing effect of morin was mainly due to its effects on OCs. Morin decreased TRAP-positive MNCs at high concentrations. Consistent with these findings, morin reduced OC survival by enhancing apoptosis, although it did not affect the differentiation of OCs. Moreover, morin reduced the surface area, maximum diameter, and fusion index of OCs that were increased by LPS in vitro, suggesting that morin attenuated the function of OCs. In line with these findings, morin inhibited bone resorption activity in dentine slices. This was attributed to a decrease in OC-specific cytoskeletal reorganization, which is critical in bone resorption. In agreement with our results, the protective effects of morin on osteoporosis have been reported in glucocorticoid-induced osteoporosis via osteogenic effects [27] and in ovariectomy-induced bone loss via acting in OCs by suppressing mitogen-activated protein kinase, NF-κB, and calcium pathways [37]. Taken together, these results indicated that morin decreased the activity and number of OCs, thereby protecting mice from inflammatory bone loss.

The plasma level of morin was reported to be ~1.4 μg/mL after 2–4 h of i.v. injection (2 mg/kg) in rats [38]. Since the injected dose (2.5–10 mg/kg) of morin in our studies could reach the plasma level of 1.75–7 μg/mL, i.e., lower than the range of our in vitro concentration (150–250 μM), assuming a similar pattern was applied, we cannot exclude a possibility that cells other than OCs could contribute to the bone-sparing effect of morin. 

To investigate the molecular mechanisms involved in the protective effect of morin in OCs, we determined whether morin affected signaling pathways associated with cytoskeletal reorganization. After 5 min of exposure, morin decreased LPS/M-CSF-induced signaling for cytoskeletal reorganization by blocking the activation of c-Src/Vav3/Rac1, an effect that has also been reported for M-CSF stimulation [39]. Given that our previous results demonstrated that ROS is responsible for the activation of c-Src in mature OCs via interaction with SHP1 [36], we hypothesized that morin may decrease ROS levels to attenuate activation of c-Src. As expected, morin dramatically decreased cytosolic ROS in mature OCs. Removal of ROS by NAC induced similar patterns to those of morin with regard to actin ring formation, as well as the level of phosphorylated c-Src. Additionally, NAC diminished the negative effects of morin on the surface area of OCs, suggesting that the inhibitory effect of morin in OCs was due to its ability to decrease ROS levels. The role of SHP1 in the inhibitory effects of morin was confirmed by silencing of SHP1 using siRNA. Knockdown of SHP1 reduced the negative effect of morin on OC surface area that was increased by LPS and M-CSF, indicating that SHP1 plays a role in mediating the effect of morin. Collectively, our findings indicated that the morin-induced decreases in OC surface area were caused by SHP1 that was converted by ROS to its active form, resulting in a blockade of cytoskeletal reorganization through the dephosphorylation of c-Src-Y416. In agreement with our results, morin has been reported to exhibit antioxidant activity in carbon tetrachloride-induced acute liver injury in mice [24] and rats [25]. Furthermore, morin has been reported to upregulate nuclear factor erythroid 2-related factor 2 (Nrf2) and hemeoxygenase-1, thereby decreasing oxidative stress in the liver and CCL4-induced hepatic fibrosis. Morin suppressed oxidative stress to reduce ifosfamide-induced neurotoxicity associated with the decrease of catalase, superoxide dismutase, and glutathione peroxidase [40].

**Figure 6:**
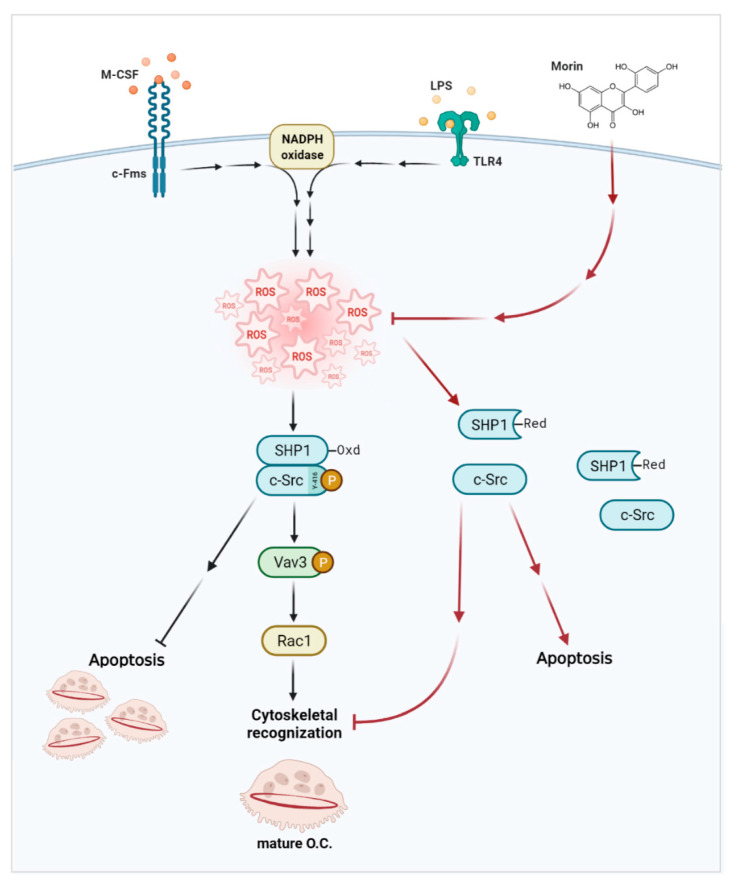
Morin decreases the number and area of OCs. LPS increased number of OCs by inhibiting apoptosis, as well as OC activity by enhancing cytoskeletal reorganization. Morin reversed both via an ROS/SHP1/c-Src axis.

Taken together, our findings demonstrated that morin granted protection against LPS-induced inflammatory bone loss by decreasing the number and area of OCs in bone without affecting bone formation (Figure 6). In vitro morin administration decreased TRAP-positive MNCs via increasing apoptosis. Morin also reduced OC area, maximum diameter, and fusion index, all of which were increased by LPS. In agreement with these findings, morin dramatically reduced OC activity with ablated cytoskeletal reorganization via a ROS/SHP1/c-Src axis. Collectively, our results demonstrated the therapeutic potential of morin against inflammatory bone loss.

## Figures and Tables

**Table 1:** Trabecular microarchitecture and biochemical markers of LPS with or without morin treatment in mice.

Parameter	Vehicle	Morin Only	LPS	LPS + Morin
BMD (mg/cm^3^)	196.7 ± 8.47	189.0 ± 12.86	150.1 ± 7.33 ^a′^	213.5 ± 11.39 ^b′′^
BV/TV (%)	16.18 ± 0.86	15.96 ± 1.21	12.07 ± 0.58 ^a′^	18.48 ± 1.21 ^b′′^
Tb.Th (μm)	70.45 ± 3.69	68.41 ± 5.04	57.88 ± 2.42 ^a^	77.39 ± 3.16 ^b′′^
Tb.Sp (μm)	348.5 ± 17.40	338.7 ± 12.19	450.9 ± 30.89 ^a^	342.5 ± 10.45 ^b′^
ALP (U/L)	48.14 ± 1.00	45.97 ± 2.28	49.54 ± 1.33	48.45 ± 1.33
OCN (ng/mL)	25.90 ± 1.18	27.85 ± 1.48	27.43 ± 0.62	27.32 ± 1.95
CTX-1 (ng/mL)	25.55 ± 1.72	24.98 ± 1.41	48.67 ± 1.81 ^a′′^	37.18 ± 2.70 ^b′^
MCP-1 (pg/mL)	143.6 ± 16.59	140.1 ± 20.55	295.9 ± 13.91 ^a′′^	209.3 ± 11.66 ^b′′^
H_2_O_2_ (μM)	42.69 ± 2.50	42.08 ± 2.37	50.31 ± 1.87 ^a^	42.36 ± 1.09 ^b′^

Vehicle (*n* = 4); morin only (dissolved in DMSO, 5 mg/kg) (*n* = 4); LPS (dissolved in PBS, 5 mg/kg) (*n* = 6); LPS + morin (*n* = 6). Data are represented as the mean ± SD. Differences between groups were analyzed by two-way ANOVA followed by Bonferroni post hoc tests to compare the effect of morin (BMD, Tb.Th, Tb.Sp, MCP-1, CTX-1, and H_2_O_2_, *p* < 0.05; BV/TV, *p* < 0.01), the effect of LPS (Tb.Sp, *p* < 0.05; CTX-1 and MCP-1, *p* < 0.001), and their interaction (Tb.Sp, CTX-1, and MCP-1, *p* < 0.05; BMD, BV/TV, and Tb.Th, *p* < 0.01). ^a^ *p* < 0.05, ^a′^ *p* < 0.01, ^a′′^ *p* < 0.001 compared with vehicle control mice’ ^b′^ *p* < 0.01, ^b′′^ *p* < 0.001 compared with LPS-injected mice.

## Data Availability

All original images and data are contained within the article.

## References

[1] Staa T.V., Geusens P., Bijlsma J.W.J., Leufkens H.G.M., Cooper C. (2006). Clinical assessment of the long-term risk of fracture in patients with rheumatoid arthritis. Arthritis Rheum. Off. J. Am. Coll. Rheumatol..

[2] Kocijan R., Englbrecht M., Haschka J., Simon D., Kleyer A., Finzel S., Kraus S., Resch H., Muschitz C., Engelke K. (2015). Quantitative and qualitative changes of bone in psoriasis and psoriatic arthritis patients. J. Bone Miner. Res..

[3] Haschka J., Hirschmann S., Kleyer A., Englbrecht M., Faustini F., Simon D., Figueiredo C.P., Schuster L., Muschitz C., Kocijan R. (2016). High-resolution quantitative computed tomography demonstrates structural defects in cortical and trabecular bone in IBD patients. J. Crohn’s Colitis..

[4] Pasco J.A., Kotowicz M.A., Henry H.J., Nicholson G.C., Spilsbury H.J., Nicholson G.C., Spilsbury H.J., Box J.D., Schneider H.G. (2006). High-sensitivity C-reactive protein and fracture risk in elderly women. JAMA.

[5] Gravallese E.M., Harada Y., Wang J.T., Gorn A.H., Thornhill T.S., Goldring S.R. (1998). Identification of cell types responsible for bone resorption in rheumatoid arthritis and juvenile rheumatoid arthritis. Am. J. Pathol..

[6] Dewhirst F.E., Stashenko P.P., Mole J.E., Tsurumachi T. (1985). Purification and partial sequence of human osteoclast-activating factor: Identity with interleukin 1 beta. J. Immunol..

[7] Redlich K., Smolen J.S. (2012). Inflammatory bone loss: Pathogenesis and therapeutic intervention. Nat. Rev. Drug Discov..

[8] Orcel P., Feuga M., Bielakoff J., De Vemejoul M.C. (1993). Local bone injections of LPS and M-CSF increase bone resorption by different pathways in vivo in rats. Am. J. Physiol..

[9] Park H.J., Son H.J., Sul O.J., Suh J.H., Choi H.S. (2018). 4-Phenylbutyric acid protects against lipopolysaccharide-induced bone loss by modulating autophagy in osteoclasts. Biochem. Pharmacol..

[10] Park H.J., Gholam-Zadeh M., Suh J.H., Choi H.S. (2019). Lycorine attenuates autophagy in osteoclasts via an axis of mROS/TRPML1/TFEB to reduce LPS-induced bone loss. Oxid. Med. Cell. Longev..

[11] Park H.J., Noh A.L., Kang J.H., Sim J.S., Lee D.S., Yim M. (2015). Peroxiredoxin II negatively regulates lipopolysaccharide-induced osteoclast formation and bone loss via JNK and STAT3. Antioxid. Redox Signal..

[12] Sul O.J., Park H.J., Son H.J., Choi H.S. (2017). Lipopolysaccharide (LPS)-induced autophagy is responsible for enhanced osteoclastogenesis. Mol. Cells.

[13] Sul O.J., Rajasekaran M., Park H.J., Suh J.H., Choi H.S. (2019). MicroRNA-29b enhances osteoclast survival by targeting Bcl2-modifying factor after lipopolysaccharide stimulation. Oxid. Med. Cell. Longev..

[14] Sul O.J., Sung Y.B., Rajasekaran M., Ke K., Yu R., Back S.Y., Choi H.S. (2018). MicroRNA-155 induces autophagy in osteoclasts by targeting transforming growth factor β-activated kinase 1-binding protein 2 upon lipopolysaccharide stimulation. Bone.

[15] Blangy A., Bompard G., Guerit D., Marie P., Maurin J., Morel A., Vives V. (2020). The osteoclast cytoskeleton—Current understanding and therapeutic perspectives for osteoporosis. J. Cell Sci..

[16] McHugh K.P., Dilke K.H., Zheng M.H., Namba N., Lam J., Novack D., Feng X., Ross F.P., Hynes R.O., Teitelbaum S.L. (2000). Mice lacking beta3 integrins are osteosclerotic because of dysfunctional osteoclasts. J. Clin. Invest.

[17] Destaing O., Sanjay A., Itzstein C., Horne W.C., Toomre D., Camilli P.D., Baron R. (2008). The tyrosine kinase activity of c-Src regulates actin dynamics and organization of podosomes in osteoclasts. Mol. Biol. Cell.

[18] Soriano P., Montgomery C., Geske R., Bradley A. (1991). Targeted disruption of the c-src proto-oncogene leads to osteopetrosis in mice. Cell.

[19] Faccio R., Novack D.V., Zallone A., Ross F.P., Teitelbaum S.L. (2003). Dynamic changes in the osteoclast cytoskeleton in response to growth factors and cell attachment are controlled by beta3 integrin. J. Cell Biol..

[20] Croke M., Ross F.P., Korhonen M., Williams D.A., Zou W., Teitelbaum S.L. (2011). Rac deletion in osteoclasts causes severe osteopetrosis. J. Cell Sci..

[21] Faccio R., Teitelbaum S.L., Fujikawa K., Chappel J., Zallone A., Tybulewicz V.L., Ross F.P., Swat W. (2005). Vav3 regulates osteoclast function and bone mass. Nat. Med..

[22] Caselli A., Cirri P., Santi A., Paoli P. (2016). Morin: A promising natural drug. Curr. Med. Chem..

[23] Jovanovic S.V., Simic M.G. (2000). Antioxidants in nutrition. Ann. N. Y. Acad. Sci..

[24] Li X., Yao Q., Huang J., Jin Q., Xu B., Chen F., Tu C. (2019). Morin hydrate inhibits TREM-1/TLR4-mediated inflammatory response in macrophages and protects against carbon tetrachloride-induced acute liver injury in mice. Front. Pharmacol..

[25] Sang L., Wang X.M., Xu D.Y., Sang L.X., Han Y., Jiang L.Y. (2017). Morin enhances hepatic Nrf2 expression in a liver fibrosis rat model. World J. Gastroenterol..

[26] Verma V.K., Malik S., Mutneja E., Sahu A.K., Bhatia J., Arya D.S. (2020). Attenuation of ROS-mediated myocardial ischemia-reperfusion injury by morin via regulation of RISK/SAPK pathways. Pharmacol. Rep..

[27] Wang C., Wan X., Li Y., Zhang H., Zhang L. (2018). Morin protects glucocorticoid-induced osteoporosis through regulating the mitogen-activated protein kinase signaling pathway. J. Nat. Med..

[28] Gao Y., Grassi F., Ryan M.R., Terauchi M., Page K., Yang X., Weitzmann M.N., Pacific R. (2007). IFN-gamma stimulates osteoclast formation and bone loss in vivo via antigen-driven T cell activation. J. Clin. Invest.

[29] Phan T.V., Sul O.J., Ke K., Lee M.H., Kim W.K., Cho Y.S., Kim H.J., Kim S.Y., Chung H.T., Choi H.S. (2013). Carbon monoxide protects against ovariectomy-induced bone loss by inhibiting osteoclastogenesis. Biochem. Pharmacol..

[30] Okayasu M., Nakayachi M., Hayashida C., Ito J., Kaneda T., Masuhara M., Suda N., Sato T., Hakeda Y. (2012). Low-density lipoprotein receptor deficiency causes impaired osteoclastogenesis and increased bone mass in mice because of defect in osteoclastic cell-cell fusion. J. Biol. Chem..

[31] Jimi E., Akiyama S., Tsurukai T., Okahashi N., Kobayashi K., Udagawa N., Nishihara T., Takahashi N., Suda T. (1999). Osteoclast differentiation factor acts as a multifunctional regulator in murine osteoclast differentiation and function. J. Immunol..

[32] Kim H.J., Zhao H., Kitaura H., Bhattacharyya S., Brewer J.A., Muglia L.J., Ross F.P., Teitelbaum S.L. (2006). Glucocorticoids suppress bone formation via the osteoclast. J. Clin. Invest.

[33] Giannoni E., Buricchi F., Raugei G., Ramponi G., Chiarugi P. (2005). Intracellular reactive oxygen species activate Src tyrosine kinase during cell adhesion and anchorage-dependent cell growth. Mol. Cell. Biol..

[34] Teitelbaum S.L. (2011). The osteoclast and its unique cytoskeleton. Ann. N. Y. Acad. Sci..

[35] Shalev M., Elson A. (2019). The roles of protein tyrosine phosphatases in bone-resorbing osteoclasts. Biochim. Biophys. Acta Mol. Cell Res..

[36] Ke K., Sul O.J., Choi E.K., Safdar A.M., Kim E.S., Choi H.S. (2014). Reactive oxygen species induce the association of SHP-1 with -Src and the oxidation of both to enhance osteoclast survival. Am. J. Physiol. Endocrinol. Metab..

[37] Shi Y., Ye L., Shen S., Qian T., Pan Y., Jiang Y., Lin J., Liu C., Wu Y., Wang X. (2021). Morin attenuates osteoclast formation and function by suppressing the NF-κB, MAPK and calcium signalling pathways. Phytother. Res..

[38] Li J., Yang Y., Lu L., Ma Q., Zhang J. (2018). Preparation, characterization and systemic application of self-assembled hydroxyethyl starch nanoparticles-loaded flavonoid Morin for hyperuricemia therapy. Int. J. Nanomed..

[39] Faccico R., Takeshita S., Zallone A., Ross F.P., Teitelbaum S.L. (2003). c-Fms and the alphavbeta3 integrin collaborate during osteoclast differentiation. J. Clin. Invest.

[40] Celik H., Kucukler S., Comaklı S., Özdemir S., Caglayan C., Yardım A., Kandemir F.M. (2020). Morin attenuates ifosfamide-induced neurotoxicity in rats via suppression of oxidative stress, neuroinflammation and neuronal apoptosis. Neurotoxicology.

